# Clinical Determinants Associated With Viral Load Count Among Adult TB/HIV Co-Infected Patients: A Linear Mixed-Effects Model Analysis

**DOI:** 10.1155/av/4514560

**Published:** 2025-08-11

**Authors:** Nurye Seid Muhie, Habib Mohammed Yimam, Awoke Seyoum Tegegne, Abdela Assefa Bekele

**Affiliations:** ^1^Department of Statistics, Mekdela Amba University, Tulu Awulia, Ethiopia; ^2^Department of Statistics, Bahir Dar University, Bahir Dar, Ethiopia; ^3^Department of Statistics, Assosa University, Asosa, Ethiopia

**Keywords:** clinical determinants, co-infected, HIV/AIDS, linear mixed, repeated measure, tuberculosis, viral load

## Abstract

HIV is a major cause of tuberculosis. The objective of current study was to isolate clinical determinants associated with viral load count among adult TB/HIV co-infected patients. This study was done at the University of Gondar Comprehensive Specialized Hospital from March 2017 to March 2022. In this study, linear mixed-effects models were used for repeated measure viral load count. Results from the analysis show that baseline viral load count (*β* = 465.1,  *p* value = 0.0026), hemoglobin levels (*β* = −493.5,  *p* value = 0.0107), CD4 cell count (*β* = −38.2,  *p* value = 0.0027), CPT (*β* = −326.8,  *p* value = 0.0363), functional status (*β* = 416.0,  *p* value = 0.0059), OCC (*β* = 123.0,  *p* value = 0.0028), tuberculosis type (*β* = 430.3,  *p* value = 0.0336), platelet cell count (*β* = −2.5,  *p* − *value* = 0.0005), lymphocyte count (*β* = −7.9,  *p* value = 0.0219), and visit time (*β* = −2.2,  *p* value = 0.001) were clinical determinants that affected repeated measure viral load count at a 5% level of significance. The study examined clinical determinants of repeated measure viral load count among TB/HIV co-infected patients. The clinical determinants like hemoglobin levels ≥ 11 g/dL, CD4 cell count ≥ 200 cell/mm^3^, CPT drug users, and platelet cell count, lymphocyte count, and visit time were decreased viral load count. Inversely, baseline viral load count (≥ 10,000 copies/mL), bedridden patients, patients with OCC, and those with extrapulmonary tuberculosis had a higher viral load count. Extensive monitoring and counseling can be beneficial for patients with hemoglobin, CD4 cell count, CPT, platelet cell count, lymphocyte count, visit time, baseline viral load count, and functional status, OCC, and TB type. Finally, further studies should be done in order to address major clinical determinants and enhance continuous follow-ups, monitor TB/HIV progression, and improve the life expectancy of patients living with TB/HIV.

## 1. Introduction

Human immunodeficiency virus (HIV) is a major cause of tuberculosis (TB). Being infected with both HIV/acquired immunodeficiency syndrome (AIDS) and TB is called TB/HIV co-infection [1, 2]. TB is the most prevalent major opportunistic illness in individuals living with HIV, and it is considered one of the world's double burden diseases. TB is the primary cause of death among HIV patients, accounting for one-third of all AIDS-related deaths [3, 4]. According to World Health Organization (WHO), 10 million people became ill with TB, and 1.6 million died from the disease, of which 26% were caused by HIV and TB co-infection [5].

An increase in HIV viral load count, increased risk of opportunistic infections (OIs), and mortality [6] in patients leads to fast development of TB [7]. Despite receiving antiretroviral medication, there is a higher risk of contracting, reactivating, and reinfecting with TB along the course of HIV [8]. Overall, HIV-infected individuals have a risk of developing TB that is between 20 and 37 times greater than that of HIV-uninfected people, and this risk persists throughout the course of HIV disease [9, 10].

According to a report from the WHO, around 10 million individuals would contract TB in 2019. Globally, around 1.7 billion people have become infected with TB, with 5%–10% developing active TB disease during their lifetime. The second-highest TB infection (25%) is found in Africa, next to Southeast Asia (44%). The highest prevalence of TB among HIV patients occurs in Africa [11, 12]. Ethiopia is one of the world's 30 high TB and HIV burden countries, with an incidence of 164 and 17 cases per 100,000 people, respectively [13, 14].

Different studies have been done in different areas related to viral load count; some of them are joint modeling of incidence of TB and change in viral load over time [15], determinants of viral load suppression among HIV-positive adults [16], factors linked to recent unsuppressed viral load in HIV-1-infected patients [17], machine learning to predict viral load failure [18], and monitoring plasma viral load and CD4 count. However, there is a scarcity of previous studies, as some mentioned above, among TB/HIV co-infected clients' viral load count in terms of the longitudinal linear mixed-effect model (LMEM). Therefore, the objective of this study was to isolate clinical determinants associated with viral load count among adult TB/HIV co-infected patients. This study was the first retrospective population-based study using repeated measure viral load data and can assist in understanding the TB/HIV co-infected clients. Consequently, filling in this knowledge gap makes sense in order to assist public health planners, policy makers, and implementers to developing effective involvement actions.

## 2. Materials and Methods

### 2.1. Study Area

This study was done at the University of Gondar Comprehensive Specialized Hospital (UGCSH).

### 2.2. Study Design

The study design for this study was an institution-based retrospective follow-up study.

### 2.3. Study Period

The study was conducted between March 2017 and March 2022.

### 2.4. Inclusion Criteria and Exclusion Criteria

The inclusion criteria for this study were TB/HIV co-infected patients whose age was greater than or equal to 15 years who received ART, patients who had at least two follow-ups periods of repeated measure viral load counts, and patients whose study period was between March 2017 and March 2022. Patients who were lost to follow-up, dropped, transferred out to the other nearest clinic, and died due to other causes were considered as exclusion criteria. Patients who had only one measurement time for variable of interest, patients who had only TB or HIV, and patients who had follow-up outside March 2017 and 2022 were excluded.

### 2.5. Study Population and Sample Size

**Figure 1:**
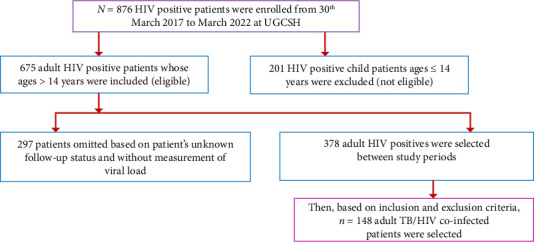
Identification of study participants.

Out of 876 HIV-positive individuals, 148 TB/HIV co-infected patients were included based on inclusion and exclusion criteria. Then, in this study, 148 TB/HIV co-infected study participants were included (Figure 1).

### 2.6. Study Variable

Repeated measure viral load counts of co-infected patients were considered as study variables. Based on the health status of patients, the number of times for the outcome variable was approximately measured within every 12 months starting from the baseline months, 12, 24, 36, 48, and 60 months.

### 2.7. Operational Definition

#### 2.7.1. Viral Load

Viral load is the amount of HIV in a person's blood. When a person is affected with HIV, the more copies of the virus there are, the higher a person's viral load. Viral load test results help healthcare providers follow what's happening with infection, know if HIV treatment is working, and decide on treatment choices [1, 5, 11], and [12].

### 2.8. Independent Variables

In this study, baseline viral load count, CD4 count in cells/mm^3^, hemoglobin level in g/dL, weight in kg, body mass index (BMI), WHO clinical stage, treatment adherence, functional status, OIs other than TB, other comorbid conditions (OCC), ART regimen, isoniazid acid hydrazide (INH), cotrimoxazole prophylactic therapy (CPT), hematocrit in %, white blood cell (WBC) in 10^3^/μL, red blood cell (RBC) in 10^6^/μL, platelet in 10^3^/μL, lymphocyte in %, monocyte in %, types of TB, and visit time were considered as independent variables.

### 2.9. LMEM

LMEMs are used for regression analyses involving dependent data that arise when working with longitudinal and other study designs in which multiple observations are made on each subject. Then, a model with both fixed effects and random effects is called a mixed effects model [19, 20]. The random effects structure reflects understanding of where to expect variance and how nested data will interact with that variance. The assumptions are random and fixed effects. The random effects assumption states that individual unobserved heterogeneity is uncorrelated with the independent variables.

The model includes both random intercepts and a random slope model. Random intercept models assign a group-specific value to each response in the group. Random slopes models, where the responses in a group follow a (conditional) mean trajectory that is linear in the observed covariates, with the slopes (and possibly intercepts) varying by group. These random terms use covariate variables to determine the conditional mean of each observation. Therefore, the LMEM model can be formulated as follows:

(1) $$\begin{array}{c} y_{ki}\left(t\right)=X_{ki}^{\prime }\left(t\right)\beta_{k}+Z_{ki}^{\prime }\left(t\right)b_{ki}+\varepsilon_{ki}\left(t\right)=m_{ki}\left(t\right){+\varepsilon }_{ki}\left(t\right), \end{array}$$

where *β*_*k*_ is the corresponding vector of the fixed effects, *X*_*ki*_′ (*t*) is the design matrix of fixed effects, including time effects and baseline covariates, *b*_*ki*_ ~ *N*(0, ψ ) is the corresponding vector of random effects, *Z*_*ki*_′(*t*) is the design matrix of (size *n* × *q*) random effects covariates, *ε*_*ki*_(*t*) is the corresponding measurement error term and distributed as *ε*_*ki*_(*t*) ~  *N*(0, *δ*_*K*_^2^*I*_*ni*_) and ⁣*m*_*ki*_(*t*) = *X*_*ki*_(*t*)*β* + *Z*_*ki*_(*t*)*b*_*ki*_ is the trajectory function.

In this study, we cannot use any variable selection strategy/methods. Because, based on previous literature, we included all important determinants under the model.

### 2.10. Missing Data Handling

Missing or incomplete data can lead to unreliable insights and poor decision-making, making it essential for anyone involved in data collection and analysis to know how to tackle these challenges [19, 21]. Then, to manage missing or incomplete data, we used multiple imputation methods as missing data handling.

### 2.11. Selection of Covariance Structures

By considering the smallest AIC, BIC, and −2Log likelihood criteria, autoregressive first-order (AR(1)) covariance structures were selected.

### 2.12. Random Effect and Survival Model Selection Criteria

The model with the smallest value of AIC, BIC and −2Log likelihood was the better-fitted model to the patient's data.

### 2.13. Methods of Data Analysis

The data were analyzed using Statistical Package for Social Science (SPSS) and R software, and statistical decisions were made at a 5% level of significance.

### 2.14. Model Diagnostic Tests

Residuals are frequently used to assess model adequacy. It is also predicted to evaluate normality, and constant variance in model diagnostics of repeated measure data. Then, the assumption of constant variance was checked by plotting the residuals against the fitted values.

### 2.15. Normality of Residual Plot

A departure from the normality of the residuals is indicated by significant deviations from the linearity of the data or nonsymmetric scales.

## 3. Results

### 3.1. Baseline Clinical Characteristics of Patients

Beyond 148 co-infected participants, 44.9% of extrapulmonary TB had an unsuppressed viral load count. Half of the patients (45.6%) with hemoglobin levels < 11 g/dL had unsuppressed viral load counts. Likewise, more than one-half of patients (54.5%) were affected by weight < 50 kg, of which 26.5% had an unsuppressed viral load count. The other clinical determinants were similar expression, as stated above. In addition, the *p* value < 0.25 under Pearson chi-square with *p* values indicated for each univariable covariate is a high chance of selecting for multivariable covariates (Table 1).

The maximum values of hematocrit, WBC, RBC, platelet cell count, lymphocyte count, and monocyte count were 54, 11, 9, 583, 69.1, and 13.4, respectively. Similarly, the standard deviation of hematocrit, WBC, RBC, platelet cell count, lymphocyte count, and monocyte count were 4.7, 0.3, 1.11, 99.92, 12.4, and 2.4, respectively. Furthermore, the skewness value (statistics) is between 0 and 0.5, and the distribution of patient data is approximately symmetrical (Table 2).

### 3.2. Selection of Covariance Structure

In this study, the AR(1) covariance structure was selected for the repeated measure viral load count (Table 3). Then, the LMEM analysis was based on AR(1).

### 3.3. Selection of LMEM

Based on Table 4, the smallest value of AIC, BIC, and log-likelihood was random intercept and slope model. Therefore, the best model for this study was random intercept and slope model.

### 3.4. Normality Assumption of Viral Load Count

**Figure 2:**
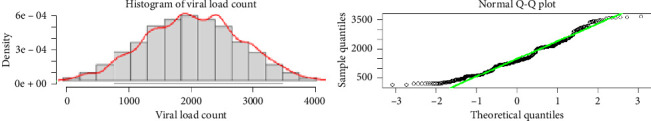
Viral load count histogram plot and Q-Q plot.

Before LMEM analysis, the normality assumption of viral load count ought to be checked. In this study, we used quantile-quantile plot (Q-Q plot) and histogram plot in order to check the assumption of normality for repeated measures viral load count. Graphical plots show that the data's normality assumption appears to be satisfied (Figure 2).

### 3.5. Interpretation of Significant Determinants

From LMEM results, baseline viral load count, hemoglobin, CD4 cell count, CPT, functional status, OCC, TB type, platelet cell count, lymphocyte count, and visit time were clinical determinants that affected repeated measure viral load count at a 5% level of significance (Table 5).

Table 5 reveals that baseline viral load count had significant determinants on repeated measure viral load count. Therefore, the average viral load count of baseline viral load count ≥ 10,000 copies/mL had increased by 465.1 copies/mL as compared to baseline viral load count < 10,000 copies/mL (*β* = 465.1,  *p* value = 0.0026). On the other side, the other covariates constant, the average viral load count of hemoglobin ≥ 11 g/dL was decreased by 493.5 copies/mL than hemoglobin < 11 g/dL (*β* = −493.5,  *p* value = 0.0107).

Relating CD4 cell count ≥ with < 200 cells/mm^3^, the average viral load count of CD4 cell count (≥ 200 cells/mm^3^) was decreased by 38.2 copies/mL, given the other determinants constant (*β* = −38.2,  *p* value = 0.0027). Correspondingly, comparing CPT drug users with nonusers co-infected patients, the average virus load for CPT drug user patients was lower than that of non-CPT users (*β* = −326.8,  *p* value = 0.0363).

The average viral load count for bedridden patients was increased by 416.0 copies/mL as compared to working patients, given the other determinants constant (*β* = 416.0,  *p* value = 0.0059). In the other expression, the average virus of co-infected patients with OCC was increased by 123 copies/mL as compared to patients without OCC, given the other determinants constant (*β* = 123.0,  *p* value = 0.0028). In similar ways, the average viral load count of extrapulmonary patients was increased by 430.3 copies/mL as compared to pulmonary patients, given the other determinants constant (*β* = 430.3,  *p* value = 0.0336).

In other expressions, the platelet cell count of patients increased by a 10^3^/μL, the average viral load count was decreased by 2.5 copies/mL (*β* = −2.5,  *p* value = 0.0005). In the same way, the lymphocyte count of patients increased by *a* %, the average viral load count was decreased by 7.9 copies/mL (*β* = −7.9,  *p* value = 0.0219).

Similarly, visit time of patients increased by a month, and the average viral load count was decreased by 2.2 copies/mL (*β* = −2.2,  *p* value = 0.001).

### 3.6. Model Diagnostic Tests

**Figure 3:**
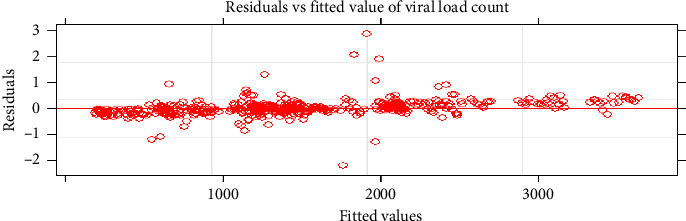
Residuals versus fitted values plot for repeated measure viral load count.

Plots of residuals against fitted values showed that the residuals trended very close to the fitted line (concentrated around zero), and there was no systematic pattern of these residuals. We can conclude that the assumption of constant variance had satisfied for repeated measure viral load counts (Figure 3).

### 3.7. Normality of Residual Plot

**Figure 4:**
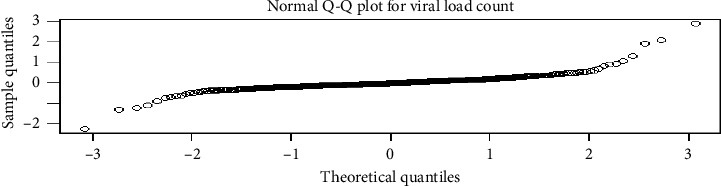
Normal Q-Q plot for residuals of repeated measure viral load count.

The residuals' normality was assessed by a Q-Q plot. Then, in Figure 4, the assumption of residuals' normality has been satisfied among co-infected patients.

## 4. Discussions

The average viral load count for baseline viral load count ≥ 10,000 was 465.1 copies/mL higher than the baseline viral load count < 10,000 copies/mL. This implied patients whose baseline viral load count was ≥ 10,000 copies/mL were higher, which leads to high viral load counts. The result of this study is supported by previous literature [22]. Conversely, this idea contradicts previous literature [15]. This discrepancy could be due to theoretical, methodological, or population differences.

Hemoglobin level has a great association with the viral load count. This result indicates that a higher value of hemoglobin leads to lower levels of viral load count. This finding is in line with a study done in Ethiopia [16]. The possible cause for this likeness might be comparable methodological analysis.

The current study also revealed that baseline CD4 cell count was another significant factor associated with viral load count. Patients with better immunity levels (≥ 200 cells/mm^3^) have lower expected levels of viral load count as compared to < 200 cells/mm^3^ [16]. This result expressed that there is a controversial relationship between patients' immune systems [22] and viral load counts [23]. The result of this study is in line with former literature. This nearness could be due to a similar methodology.

Adult CPT drug users and co-infected patients had considerably lower viral load counts than non-CPT users. This result is in line with [24, 25]. In contrast, co-infected individuals with OCC had a larger quantity of viral load concentration than patients with OCC [26]. This result indicates co-infected patients have a higher risk of death [27] due to an increment of virus concentration and a decrement of virus suppression [28].

This study also indicates that bedridden co-infected patients have higher viral load counts than working patients [29]. As a result, patients may be unable to work or care for themselves, require varying amounts of assistance from family members or others, and live at home as a result of disease evolution. Our finding is supported by previous literature [30].

The average viral load count increases with the decreasing rate of platelet cell count. This study is contradicted by previous studies [31]. The potential causes of these discrepancies may vary depending on the study area and time, sample size, and methodology used. As a result, it requires additional examination.

TB status of co-infected patients has great importance for the variation of viral load count. The one logical reason is that co-infected patients with extrapulmonary TB have a loss of appetite and do not synthesize energy, which leads to a reduction of body immunity [6] and the development of diseases [8], like OIs [32]. As a result, patients' viral load count increases with visit time [9]. The possible intention for this similarity might be due to the same expressions of statistical methodology.

Visit time of patients shows a significant decrement variation for viral load count [33]. This implies, patients measure the virus concentration at different times (approximately 12-month intervals) by health professionals [34], significantly leading to a decrement of viral load count [35]. Due to decrement variations of the virus [36], the average number of individuals caused high viral load suppression [37] or a lower death rate [38]. The result of this study was also supported with previous literature [39]. The possible intention for this similarity might be due to the same predictors of visit time [40].

## 5. Conclusions and Recommendations

The study examined the clinical determinants of viral load count results of TB/HIV co-infected patients in the UGCSH. The determinants like hemoglobin ≥ 11 g/dL, CD4 cell count ≥ 200 cell/mm^3^, CPT drug users, and platelet cell count, lymphocyte count, and visit time were decreased viral load count. Inversely, baseline viral load count (≥ 10,000 copies/mL), bedridden patients, patients with OCC, and patients with extrapulmonary TB had a higher amount of viral load count. Clinically, the findings of this study contribute to extending patient survival and guiding treatment interventions. Therefore, extensive monitoring and counseling can be beneficial for patients with hemoglobin, CD4 cell count, CPT, platelet cell count, lymphocyte count, visit time, baseline viral load count, and functional status, OCC, and TB type. Finally, further studies should be consider in order to address major clinical determinants and enhance continuous follow-ups, monitor TB/HIV progression, and improve the life expectancy of patients living with TB/HIV.

## Figures and Tables

**Table 1:** Baseline clinical characteristics of TB/HIV co-infected patients.

Variables	Categories	Unsuppressed viral load (%)	Censored (%)	Total (%)	Pearson chi-square	*p* value
Types of TB	Pulmonary	8 (13.6)	51 (86.4)	59 (39.9)	15.948	0.001
	Extrapulmonary	40 (44.9)	49 (55.1)	89 (60.1)		

Hemoglobin	< 11 g/dL	36 (45.6)	43 (54.4)	79 (53.4)	13.345	0.001
	≥ 11 g/dL	12 (17.4)	57 (82.6)	69 (46.6)		

Weight	< 50 kg	30 (37.5)	50 (62.5)	80 (54.1)	2.040	0.105
	≥ 50 kg	18 (26.5)	50 (73.5)	68 (45.9)		

Baseline viral load	< 10,000 copies/mL	15 (17.9)	69 (82.1)	84 (56.8)	18.831	0.001
	≥ 10,000 copies/mL	33 (51.6)	31 (48.4)	64 (43.2)		

Treatment adherences	Poor	16 (36.4)	28 (63.4)	44 (29.7)	0.629	0.370
	Fair	21 (32.3)	44 (67.7)	65 (43.9)		
	Good	11 (28.2)	28 (71.8)	39 (26.4)		

WHO clinical stage	Stage-I	10 (6.8)	30 (20.3)	40 (27.0)	20.836	0.001
	Stage-II	15 (31.3)	33 (68.8)	48 (32.4)		
	Stage-III	25 (53.2)	22 (46.8)	47 (31.8)		
	Stage-IV	5 (38.5)	8 (61.5)	13 (8.8)		

Baseline CD4 cell	< 200 cells/mm^3^	33 (47.1)	37 (52.9)	70 (47.3)	13.116	0.001
	≥ 200 cells/mm^3^	15 (19.2)	63 (80.8)	78 (52.7)		

OIs	No	28 (24.8)	85 (75.2)	113 (76.4)	12.773	0.001
	Yes	20 (57.1)	15 (42.9)	35 (23.6)		

INH	No	12 (15.4)	66 (84.6)	78 (52.7)	21.871	0.001
	Yes	36 (51.4)	34 (48.6)	70 (47.3)		

CPT	No	10 (12.0)	73 (88.0)	83 (56.1)	35.834	0.001
	Yes	38 (58.5)	31 (41.5)	65 (43.9)		

ART regiment	1d	14 (31.1)	31 (68.9)	45 (30.4)	3.148	0.369
	1c	12 (24.5)	37 (75.5)	49 (33.1)		
	1e	9 (40.9)	13 (59.1)	22 (14.9)		
	Others	13 (40.6)	19 (59.4)	32 (21.6)		

Functional status	Working	19 (22.4)	66 (77.6)	85 (57.4)	11.306	0.004
	Ambulatory	13 (38.2)	21 (61.8)	34 (23.0)		
	Bedridden	16 (55.2)	13 (44.8)	29 (19.6)		

BMI	< 18.5 kg/m^2^	26 (32.1)	55 (67.9)	81 (54.7)	3.008	0.022
	18.5–24.9 kg/m^2^	17 (40.5)	25 (59.5)	42 (28.4)		
	≥ 25 kg/m^2^	5 (20.0)	20 (80.0)	25 (16.9)		

OCC	No	32 (27.4)	85 (72.6)	117 (79.1)	6.583	0.011
	Yes	16 (51.6)	15 (48.4)	31 (20.9)		

*Note:* g/dL indicates gram per deciliter, kg/m^2^ indicates kilogram per meter square, copies/mL indicates copies per milliliter, 1 day refers to ART treatment of AZT-3TC-EFV, 1c refers to ART treatment of AZT-3TC-NVP, 1e refers to TDF-3TC-EFV and other means other ART treatments, like 1j (TDF + 3 TC + DTG) and 1f (TDF + 3 TC + NVP).

**Table 2:** Descriptive statistics for clinical variables.

Continuous variables	Minimum	Maximum	Mean	Standard deviation	Skewness	
					Statistic	Std. error
Hematocrit in %	27.90	53.60	38.1595	4.73104	0.463	0.199
WBC in 10^3^/μL	2.60	10.90	5.9074	1.70816	0.352	
RBC in 10^6^/μL	2.00	8.10	4.0813	1.12154	0.080	
Platelet in 10^3^/μL	20	566	265.09	99.187	0.411	
Lymphocyte in %	21.0	69.1	45.196	12.3547	0.199	
Monocyte in %	2.3	55.4	6.603	12.3547	0.248	

**Table 3:** Covariance structure comparison criteria.

Covariance structure	AIC	BIC	Log-likelihood
AR (1)	5735.543	5875.03	−2833.771
UN	5752.048	5891.535	−2842.024
CS	5750.104	5889.591	−2841.052

*Note:* AR (1) represents autoregressive first order, UN indicates unstructured.

Abbreviation: CS, compound symmetry.

**Table 4:** Linear mixed effect model comparison criteria.

Models	AIC	BIC	Log-likelihood
Random intercept	6354.866	6371.519	−3173.433
Random slope	6513.09	6538.057	−3250.545
Random intercept and slope	5748.104	5883.488	−2841.052

**Table 5:** Linear mixed-effect model results for viral load count.

Variables	Categories	Estimate (*β*)	Standard error	*p* value
Intercept	—	1086.153	943.705	0.0250^∗^

Baseline viral load count (ref = < 10,000 copies/mL)	≥ 10,000 copies/mL	465.1111	152.992	0.0026^∗^

Hemoglobin (ref = < 11 g/dL)	≥ 11 g/dL	−493.466	308.503	0.0107^∗^

Adherence (ref = poor)	Fair	−175.8583	190.5431	0.3058
	Good	48.8828	207.7299	0.8143

WHO (ref = stage-I)	Stage-II	129.2140	175.7851	0.4629
	Stage-III	−37.4238	199.7453	0.8515
	Stage-IV	259.0464	285.0798	0.3064

CD4 cell (Ref = < 200 cells/mm^3^)	≥ 200 cells/mm^3^	−38.1656	329.039	0.0027^∗^

OIs (ref = no)	Yes	25.0131	297.0715	0.3030

INH (ref = No)	Yes	54.7061	187.6115	0.7710

CPT	Yes	−326.7697	155.3603	0.0363^∗^

ART regiment (Ref = 1day)	1c	−61.0715	172.8141	0.7240
	1e	112.9706	193.0467	0.5588
	Others	7.2122	200.0455	0.9713

Functional status (Ref = working)	Ambulatory	−60.2005	182.0894	0.7412
	Bedridden	416.0007	219.3561	0.0059^∗^

Weight (ref = < 50 kg)	≥ 50 kg	−212.4409	153.7031	0.1679

BMI (ref = < 18.5 kg/m^2^)	18.5–24.9 kg/m^2^	−83.8169	172.3190	0.6270
	≥ 25 kg/m^2^	−187.5525	215.4093	0.3846

OCC (ref = no)	Yes	123.0448	273.2443	0.0028^∗^

TB type (ref = pulmonary)	Extrapulmonary	430.2767	201.5426	0.0336^∗^

Hematocrit in %	—	1.0774	19.4602	0.9559

WBC in 10^3^/μL	—	17.9035	37.5654	0.6340

RBC in 10^6^/μL	—	−68.3277	72.5133	0.3468

Platelet in 10^3^/μL	—	−2.4840	0.7019	0.0005^∗^

Monocyte in %	—	−13.5370	16.8024	0.4211

Lymphocyte in %	—	−7.9541	6.4649	0.0219^∗^

Visit time	—	−2.1586	0.2816	0.001^∗^

*Note:* g/dL indicates gram per deciliter, kg/m^2^ indicates kilogram per meter square, copies/mL indicates copies per milliliter, 1 day refers to ART treatment of AZT-3TC-EFV, 1c refers to ART treatment of AZT-3TC-NVP, 1e refers to TDF-3TC-EFV and other means other ART treatments, like 1j (TDF + 3 TC + DTG) and 1f (TDF+3 TC + NVP).

^∗^Statistically significant at 5% level of confidence.

## Data Availability

The data that support the findings of this study are available from the corresponding author upon reasonable request.

## Nomenclature

AIC: Akaike information criteria
AR(1): Autoregressive first order
ART: Antiretroviral therapy
BIC: Bayesian information criterion
BMI: Body mass index
CD4: Cluster differentiation 4
CPT: Cotrimoxazole preventive therapy
CS: Compound symmetry
HIV: Human immunodeficiency virus
INH: Isoniazid acid hydrazide
LMEM: Linear mixed-effect model
OCC: Other comorbid condition
OIs: Opportunistic infections
RBC: Red blood cell
TB: Tuberculosis
WBC: White blood cell
UN: Unstructured
WHO: World Health Organization

## Appendix

Code Availability: All codes organized in this study were available from the corresponding author on reasonable request.

## Supporting Information

The additional information in this article is provided in the supporting information for review and production.
