# Supplementary material for: Clinical Determinants Associated With Viral Load Count Among Adult TB/HIV Co-Infected Patients: A Linear Mixed-Effects Model Analysis
Source: Adv Virol. 2025 Aug 11;2025:4514560. doi: 10.1155/av/4514560 (PMC12360880; doi:10.1155/av/4514560)
Supplement: Supporting Information — Additional supporting information can be found online in the Supporting Information section. [file 4514560.f1.docx]

**R packages as sample code.**

library(tcltk)

library(tkrplot)

library(lattice)

library(MASS)

library (graphics)

library(ggplot2)

library(ConvergenceConcepts)

attach(normR)

library(ggpubr)

install.packages("dplyr")

install.packages("devtools")

library(dplyr)

library(devtools)

***********Histogram for viral load count**

hist(vlc_last_5000$VLC, probability=T, main="Histogram of viral load count

",xlab="viral load count")

lines(density(vlc_last_5000$VLC),col="green",lwd=2)

**##########QQ plot##########**

qqnorm(vlc_last_5000$VLC, pch = 1)

qqline(vlc_last_5000 $ VLC,col ="green", lwd = 3)

********Covariance structure**

********For Autoregressive first order (AR1)**

model01<-lme(VLC~ hematocrit+WBC+RBC+Platlet+lymphocte+monocyte+as.factor(viralload)+…………………..+as.factor(Ois)+ vistime, data=vlc_last_5000, random=~ vistime |ID, correlation =corAR1(form =~ 1 |ID ))

********FOR COMPOUND SYMMETRY (CS)**

model011<-lme(VLC~ hematocrit+WBC+RBC+Platlet+………………………+ vistime, data=vlc_last_5000, random=~ vistime |ID, correlation =corCompSymm(form =~1|ID))

********for Unstructured (UN)**

model011<-lme(VLC~ hematocrit+WBC+RBC+Platlet+lymphocte+monocyte+……….+ vistime, data=vlc_last_5000, random=~ vistime |ID, correlation =un(form =~1|ID))

*********Linear mixed effect model comparison**

*********For Random intercept**

intmodel<-lme(VLC ~ 1, data = vlc_last_5000, random = ~ 1|ID,correlation =corAR1(form =~ 1 |ID ))

summary(intmodel)

*********For Random slope**

randomnmmu<-lme(VLC ~ vistime,data = vlc_last_5000, random = ~ vistime |ID)

summary(randomnmmu)

*********For Random intercept and slope**

mode2025d<-lme(VLC~ hematocrit+WBC+RBC+Platlet+lymphocte+…………………+ vistime, data=vlc_last_5000, random=~ vistime |ID)

*******For Linear mixed effect model analysis**

mode2025<-lme(VLC~ hematocrit+WBC+RBC+PLATLET+lymphocte+monocyte+as.factor(viralload)+………………+ vistime, data=vlc_last_5000, random=~ vistime |ID, correlation =corAR1(form =~ 1 |ID ))

******* Residuals VS fitted value**

plot(mode2025,resid(., type="p") ~ fitted(.),ylab="Residuals",main="Residuals VS fitted value for viral load count",abline=0,col="red")

plot(mode2025)

******* Residuals VS QQ Norm**

qqnorm(residuals(mode2025,type = "p"),main="Normal Q-Q Plot for Viral load count")
